# The predictive performance of criminal risk assessment tools used at sentencing: Systematic review of validation studies

**DOI:** 10.1016/j.jcrimjus.2022.101902

**Published:** 2022

**Authors:** Seena Fazel, Matthias Burghart, Thomas Fanshawe, Sharon Danielle Gil, John Monahan, Rongqin Yu

**Affiliations:** a Department of Psychiatry, University of Oxford, Oxford, UK; b Nuffield Department of Primary Care Health Sciences, University of Oxford, UK; c Leiden University Medical Centre, Leiden, the Netherlands; d School of Law, University of Virginia, USA

**Keywords:** Sentencing, Recidivism, Risk prediction, Risk assessment

## Abstract

Although risk assessment tools have been widely used to inform sentencing decisions, there is uncertainty about the extent and quality of evidence of their predictive performance when validated in new samples. Following PRISMA guidelines, we conducted a systematic review of validation studies of 11 commonly used risk assessment tools for sentencing. We identified 36 studies with 597,665 participants, among which were 27 independent validation studies with 177,711 individuals. Overall, the predictive performance of the included risk assessment tools was mixed, and ranged from poor to moderate. Tool performance was typically overestimated in studies with smaller sample sizes or studies in which tool developers were co-authors. Most studies only reported area under the curve (AUC), which ranged from 0.57 to 0.75 in independent studies with more than 500 participants. The majority did not report key performance measures, such as calibration and rates of false positives and negatives. In addition, most validation studies had a high risk of bias, partly due to inappropriate analytical approach used. We conclude that the research priority is for future investigations to address the key methodological shortcomings identified in this review, and policy makers should enable this research. More sufficiently powered independent validation studies are necessary.

## Highlights

• Predictive performance of sentencing risk tools range from poor to moderate.
• Studies with smaller sample sizes report higher AUCs.
• Reporting of key performance measures (calibration and false negatives) is lacking.

## 1 Introduction

Risk assessment tools are widely used to inform sentencing decisions for individuals convicted of crimes in many high-income countries (van Ginneken, 2019). These tools can influence decisions on whether someone receives a prison or community-based sentence, sentence length, and associated restrictions, such as electronic tagging (Monahan & Skeem, 2016). The choice between custodial and non-custodial outcomes is significant as prison is associated with many negative ramifications for accommodation, relationships, and employment (Harding, Wyse, Dobson, & Morenoff, 2014; Keene, Smoyer, & Blankenship, 2018; Western, Braga, Davis, & Sirois, 2015). Sentence length is determined by legislation and provides a range of sentence length options, which is typically based on certain individual factors, including the previous criminal history and risk of future serious offending. These tools can also assist with treatment decisions, especially for people with mental health problems, and lead to referrals to diversion services. Risk assessment tools need to be high quality because of the potential consequences for individuals in the criminal justice system and public health and safety. For people in the criminal justice system, the use of inaccurate risk assessment tools can lead to longer periods of time in custody. For public health and safety, it can lead to wasteful and poor allocation of resources if people remain in custody who could be released based on risk levels, and the consequences of repeat offending in people released without appropriate supervision and treatment. Furthermore, the wider ethical implications on public trust in criminal justice are relevant in that such tools should be transparently designed and reported, and not lead to amplification of systemic biases and also potentially mitigate against them.

Previous work reviewing these tools has combined different samples and settings, including forensic psychiatric hospitals, intra-institutional outcomes, and non-offending samples (Campbell, French, & Gendreau, 2009; Fazel, Singh, Doll, & Grann, 2012; Ramesh, Igoumenou, Vazquez Montes, & Fazel, 2018; Singh, Serper, Reinharth, & Fazel, 2011). In addition, it has combined development (also known as discovery) samples with validation ones, which does not reflect real-world performance as the development samples tend to overestimate predictive performance (Pavlou et al., 2015). Furthermore, validation studies may have been conducted by the tool developers, which can lead to authorship bias (Singh, Grann, & Fazel, 2013), and separately examining such investigations conducted by independent groups needs consideration. Finally, previous reviews have used performance measures such as correlation coefficients and effect sizes that are not informative on their own and not recommended in standard guidelines for systematic reviewing of prediction models (Moons et al., 2014). Rather, measures of discrimination (including true and false positives and negatives), and calibration (how estimated and predicted risk scores compare) are necessary for any tool to be evaluated (Collins, Reitsma, Altman, & Moons, 2015).

To address these limitations, we have conducted a systematic review of validation studies of risk assessment tools that are used to inform decision-making in the criminal justice system. Our primary outcomes were measures of predictive performance from independent validations, where tool developers are not co-authors. In so doing, we aim to provide a focused overview that will inform criminal justice and linked mental health services.

## 2 Methods

### 2.1 Protocol and registration

This systematic review was pre-registered under the Open Science Framework (OSF). The protocol can be retrieved from: https://osf.io/59szj

### 2.2 Literature search

As a first step, we identified risk assessment tools commonly used at the sentencing stage for criminal offences to estimate risk of recidivism by searching PsycINFO, Medline, and EMBASE with the following keywords: (*psychiatry* OR *forensic* OR *psychology*) AND (*sentencing* OR *sanctioning* OR *sanction* OR *violence*) AND (*recidivism* OR *re-offense*) AND *risk assessment*. A tool was considered as commonly used based on previous reviews (Desmarais, Johnson, & Singh, 2016). In addition, reference lists from related reviews and Google Scholar were hand-searched. On the basis of this, we identified 11 widely used risk assessment instruments in criminal sentencing (Table 1).

**Table 1:** Common risk assessment instruments to inform sentencing decisions

Instrument	Abbreviation	Authors
Correctional Offender Management Profiling for Alternative Sanctions	COMPAS	Northpointe Institute for Public Management (1996)
Historical Clinical Risk Management-20	HCR-20	Douglas, Hart, Webster, and Belfrage (2013)
The Indiana Risk Assessment System	IRAS	Latessa, Lovins, and Makarios (2013)
Level of Service/Case Management Inventory	LS/CMI	Andrews, Bonta, and Wormith (2004)
Level of Service Inventory-Revised	LSI-R	Andrews and Bonta (1995)
Nonviolent Risk Assessment	NVRA	Garrett, Jakubow, and Monahan (2019)
Offender Assessment System	OASys	Howard (2006)
Ohio Risk Assessment System	ORAS	Latessa, Lemke, Makarios, and Smith (2010)
Psychopathy Checklist-Revised	PCL-R	Hare (2003)
Post Conviction Risk Assessment	PCRA	Johnson, Lowenkamp, van Benschoten, and Robinson (2011)
Static-99 (revised)	Static-99(R)	Helmus, Thornton, Hanson, and Babchishin (2012)

After the initial tool selection (made on 5/3/19), the same three electronic bibliographic databases (PsycINFO, Medline, and EMBASE), Google Scholar and related reference lists were searched from their start dates until 28 February 2021 for any validation studies assessing the predictive performance of these instruments. The search terms comprised a sentencing tool's full name and acronym in combination with the keywords *predictive* OR *validation* OR *validity*.

### 2.3 Eligibility criteria

Validation studies were considered eligible for inclusion if they examined the predictive performance of one of the included tools in an adult offender sample to inform sentencing decisions, treatment and supervision planning. The main outcome was criminal reoffending, which typically reported new convictions but could include other criminal outcomes (e.g. arrests or charges) if conviction information was not reported. We excluded (i) reviews and theoretical papers, (ii) studies investigating violence or misconduct within an institution, such as prisons or hospitals, and (iii) investigations that solely reported other outcomes. No date or language restrictions were applied.

### 2.4 Study selection

Consistent with the Preferred Reporting Items for Systematic Reviews and Meta-Analyses (PRISMA; Moher et al., 2009) guidelines, two independent reviewers were involved in the study selection process. At first, SG and MB reviewed the titles and abstracts of all identified articles. Studies that were deemed eligible proceeded to the second stage and were assessed via full text screening according to the inclusion and exclusion criteria. For inclusion, a consensus had to be reached between the two reviewers. Any disagreements on study selection were resolved in consultation with SF and RY.

In addition, to maintain independence among outcome measures (Borenstein, Hedges, Higgins, & Rothstein, 2011), overlapping samples investigating the same tool were included once. The most recent studies were included. Studies that tested the performance of different sentencing risk assessment instruments in the same publication were treated independently.

### 2.5 Data extraction

Data extraction was carried out independently by MB and GS using a standardised form. Information on the following variables were collected: (1) sample demographics (population type, sample size, sex); (2) settings in which data were collected; (3) study design and procedure (measured outcome, length of follow-up, independence of authors); (4) performance estimates of the examined sentencing risk assessment tool, including measures of discrimination and calibration, and (5) interrater reliability. We contacted corresponding authors if information was lacking or needed clarification.

When articles reported separate predictive performance measures for different forms of recidivism, the outcome most closely resembling the tool's recommended outcome was chosen to ensure consistency. In case of the Static-99, for instance, sexual recidivism was preferred over any violent reoffending or any general reoffending. Duration of follow-up varied; the one closest to 5 years was selected as this was the one most commonly reported. If studies reported results from both a combined sample and smaller subsamples, the former were extracted.

### 2.6 Summary measures

To assess the predictive performance of the included risk assessment tools, we extracted measures of discrimination and calibration. Discrimination refers to an instrument's ability to differentiate between recidivists and non-recidivists, whereas calibration quantifies how well the risk prediction corresponds to the true observed risk of an individual (Cook, 2007).

The area under the receiver operating characteristic curve (AUC) is widely used as a global discrimination index (Singh, Desmarais, & Van Dorn, 2013). The AUC expresses the probability that a randomly selected reoffender scores higher on a particular risk assessment tool than a randomly selected person who did not reoffend. AUC values range from 0.5 (i.e. discrimination no better than chance) to 1.0 (i.e. perfect discrimination). Although categorical benchmarks to assess the strength of an AUC exist, these are inconsistent, will necessarily depend on how a tool is used, and a second-order systematic review has revealed large between-study variation in the thresholds applied (Singh, Desmarais, & Van Dorn, 2013), which is why we did not use AUC thresholds. Furthermore, as many included studies failed to report the 95% confidence interval (CI) of their AUC, we estimated missing CIs from the number of recidivists and non-recidivists in the sample (Hajian-Tilaki & Hanley, 2002; Hanley & McNeil, 1982). Where possible, we additionally extracted the Brier score or the E/O (expected/observed) index as a measure of calibration. The Brier ranges between 0 and 1 and quantifies the accuracy of a tool's risk prediction by averaging the squared differences between the predicted and observed outcome probabilities (Rufibach, 2010). Lower scores indicate better accuracy. The E/O index is the ratio between the expected and observed number of recidivists (Hanson, 2017). Perfect calibration is indicated by an E/O index of 1. Finally, since good reliability is a prerequisite for an instrument to be accurate (Chambers et al., 2009), we collected information on interrater reliability in form of an intraclass correlation coefficient (ICC) or Pearson correlation coefficient (*r*), and reported summary statistics if reliability investigations were replicated (i.e. k > 1).

### 2.7 Synthesis of results

As recommended by Cochrane guidelines (Macaskill, Gatsonis, Deeks, Harbord, & Takwoingi, 2010) and methodologists (Wan, Wang, Liu, & Tong, 2014), the median and interquartile range (IQR) for all performance measures were calculated and reported separately for each sentencing risk assessment tool. This was the primary analysis due to the heterogeneity in outcome definitions and follow-up periods. For our primary analysis, we only included validations from tool-independent authors (i.e. authors who were not involved in its development) and a sample size larger than 500. This was to avoid authorship bias (Singh, Desmarais, & Van Dorn, 2013) and imprecise estimates common to smaller sample sizes (Hanczar et al., 2010). The findings from the remaining non-independent studies are reported in supplementary materials. In addition, as a secondary analysis, random-effects models were used to pool AUCs. Random-effects models were used because of large heterogeneity between studies. In addition, we examined tool performance in studies where data had been specifically collected at the pre-sentence stage. Analyses were performed with the R version 4.0.2 (R Core Team, 2020) and the metafor-package (Viechtbauer, 2010).

### 2.8 Risk of bias and publication bias

The risk of bias within each study was assessed with the Prediction model Risk Of bias ASsessment Tool (PROBAST; Wolff et al., 2019). The PROBAST was developed specifically for evaluating studies of diagnostic and prognostic prediction models and provides ratings of the risk of bias on four different levels, namely: (1) participants, (2) predictors, (3) outcome, and (4) analysis. As outlined above, bias across studies was reduced by excluding articles authored by tool designers from the primary analysis. Publication bias was examined by funnel plot asymmetry using the weighted regression approach (Egger, Smith, Schneider, & Minder, 1997).

## 3 Results

Overall, we identified 36 studies with 597,665 participants (PRISMA flowchart in **Supplementary** Fig. 1), which were based in 7 countries (Table 2). Of these, 27 were independent validation studies with 177,711 participants, reporting on 7 tools: COMPAS, HCR-20, LS/CMI, LSI-R, PCL-R, PCRA, and Static-99. No eligible validation studies were identified for IRAS and NVRA. The ORAS and OASys were only validated by the developers of these tools. The most common performance statistic reported was the AUC. Out of 36 studies, two studies (Boccaccini, Rice, Helmus, Murrie, & Harris, 2017; Hanson, Lunetta, Phenix, Neeley, & Epperson, 2014) reported measures of calibration, both which assessed the Static-99: E/O ratio = 1.90 (95% CI: 1.75 to 2.07) and E/O = 1.30 (95% CI: 0.87 to 1.96), respectively.

**Table 2:** Characteristics of included articles.

	Outcome measure	N	Sample	Stage of assessment	Follow-up length	Country
*COMPAS*						
Brennan, Dieterich, and Ehret (2009)	Arrest for any offense	2328	Male and female probationers	As part of the routine processing at entry into probation agencies	4 years	USA
Farabee, Zhang, Roberts, and Yang (2010)	Arrest for any reason	25,009	Male and female parolees	As part of the case planning for parole supervision	24 months	USA
Fass, Heilbrun, DeMatteo, and Fretz (2008)	Rearrest	276	Male offenders released from assessment and treatment centres	As part of the routine assessment and classification procedure	12 months	USA
*HCR-20*						
Dahle (2006)	Violent reoffending	307	Male offenders released from prison	Data collection was done at the beginning of the sentence	10 years	Germany
Mills, Kroner, and Hemmati (2007)	Violent reoffending	83	Male offenders released from prison	As part of the psychological assessment process (within 8–12 weeks of arrival in prison) – with the purpose to identify appropriate supervision strategies	M = 4.6 years	Canada
*LS/CMI*						
Dyck, Campbell, and Wershler (2018)	Any new charge	136	Male and female community-supervised provincial offenders	As part of the case planning by probation officers – completed at intake (e.g., within the first 3 months of supervision)	M = 41.5 months	Canada
Gordon, Kelty, and Julian (2015) [male sample]	Reconviction	569	Male offenders completing a community-based order or a custodial sentence combined with a supervision period upon release	As part of the initial assessment	12 months	Australia
Gordon et al. (2015) [female sample]	Reconviction	113	Female offenders completing a community-based order or a custodial sentence combined with a supervision period upon release	As part of the initial assessment	12 months	Australia
Tsao and Chu (2021)	Any new charge	134	Male sex offenders on community supervision	As part of the risk assessment during presentencing stage to determine suitability for community supervision	M = 3.7 years	Singapore
Wormith, Hogg, and Guzzo (2015) [Non-Aboriginal sample]	Any criminal offense	24,758	Male and female offenders released from a custodial sentence or given a conditional sentence or probation	As part of the routine presentence assessment	5 years	Canada
Wormith et al. (2015) [Aboriginal sample]	Any criminal offense	1692	Male and female offenders released from a custodial sentence or given a conditional sentence or probation	As part of the routine presentence assessment	5 years	Canada
*LSI-R*						
Barnoski and Aos (2003)	Misdemeanor and felony recidivism	22,533	Male and female offenders placed in the community following a prison stay or as part of a community supervision sanction	Within 90 days of community placement with the aim to allocate more community-based resources to higher-risk offenders	24 months	USA
Dahle (2006)	Reimprisonment	307	Male offenders released from prison	Data collection was done at the beginning of the sentence	5 years	Germany
Fass et al. (2008)	Rearrest	696	Male offenders released from assessment and treatment centres	As part of the routine assessment and classification procedure	12 months	USA
Manchak, Skeem, and Douglas (2008)	Conviction of any new offense	844	Male inmates released from prison	As part of the routine prison procedure within a year of inmates' release date	12 months	USA
Ostermann and Herrschaft (2013)	Arrest for a new crime	900	Male and female parolees	As part of the routine prerelease assessment	36 months	USA
Vose, Smith, and Cullen (2013)	Any new misdemeanor or felony conviction	2849	Male and female probationers and parolees	As part of the initial assessment	M = 1385 days	USA
Watkins (2011)	Reincarceration	11,051	Male and female offenders released from incarceration	As part of the routine presentence and prerelease assessment	24 months	Australia
*OASys*						
Howard (2015)	Any violent reoffending	92,514	Male and female offenders with community sentences or a discharge from custody	As part of routine assessment for community sentence or discharge from custody	24 months	UK
Howard and Dixon (2013)	Any violent reoffending	196,493	Male and female offenders with community sentences or postcustodial supervision	As part of a presentence court report, commencing community sentence, or supervision upon release from custody	M = 27.1 months	UK
*ORAS*						
Latessa, Lux, Lugo, and Long (2017)	New conviction	10,548	Male and female offenders under probation service supervision	As part of an initial assessment process within 45 days of disposition	14 months	USA
Lovins, Latessa, May, and Lux (2018)	Any new arrest for a criminal act or revocation for technical/law violation	5482	Male and female probationers	As part of a community supervision assessment	M = 15 months	USA
*PCL-R*						
Dahle (2006)	Reimprisonment	307	Male offenders released from prison	Data collection at the beginning of sentence	5 years	Germany
Harris, Boccaccini, and Rice (2017)	Rearrest for a sexual or violent offense	658	Male sex offenders released from custody	As part of a civil commitment evaluation	M = 10.5 years	USA
Rettenberger, Rice, Harris, and Eher (2017)	New conviction for a violent offense	739	Male sex offenders released from prison	As part of a presentence screening for treatment planning	M = 6.5 years	Austria
Tsao and Chu (2021)	Any new charge	134	Male sex offenders on community supervision	As part of the risk assessment during presentencing to determine suitability for community supervision	M = 3.7 years	Singapore
Walters and Duncan (2005)	Rearrest for any offense	91	Male offenders released from custody	As part of presentence forensic evaluation	M = 60.3 months	USA
*PCRA*						
Cohen (2018)	Rearrest for any offense	5347	Male sex offenders on federal post-conviction supervision or probation	As part of routine assessment process of the federal supervision system	> 12 months	USA
Lowenkamp, Johnson, Holsinger, VanBenschoten, and Robinson (2012)	Any new arrest	51,643	Male and female offenders on probation or supervised release	As part of federal presentence report	6–12 months	USA
Luallen, Radakrishnan, and Rhodes (2016)	Rearrest for a serious offense	84,579	Male and female offenders received into federal community supervision	As part of an initial assessment at the outset of supervision	6 months	USA
Skeem and Lowenkamp (2020)	Rearrest for a violent crime	34,021	Male and female federal probationers	As part of an initial assessment when an offender entered supervision	M = 1683 days	USA
*Static-99/R*						
Allan, Dawson, and Allan (2006)	Nonviolent sexual reoffending	144	Male sex offenders released from prison	Data was based on presentence reports	M = 9.3 years	Australia
Boccaccini et al. (2017)	Arrest for a sexual offense	17,455	Male sex offenders released from custody	As part of a risk-level determination, prerelease evaluation, parole evaluation, program entry, and civil commitment screening	5 years	USA
Etzler, Eher, and Rettenberger (2020)	New conviction for a sexual offense	520	Male sex offenders released from prison	As part of a presentence screening for treatment planning	5 years	Austria
Hanson et al. (2014)	Arrest for a sexual offense	475	Male sex offenders released from the California Department of Corrections and Rehabilitation	As part of the routine assessment of the California Department of Corrections and Rehabilitation	5 years	USA
Kingston, Yates, Firestone, Babchishin, and Bradford (2008)	Any charge or conviction with a sexual offense	192	Male sex offenders released from the Royal Ottawa Hospital Sexual Behaviors Clinic	As part of the initial assessment just prior or just after sentencing	M = 11.4 years	Canada
Looman (2006)	Conviction for a sexual offense	258	Male sex offenders released from the Regional Treatment Centre Sexual Offender Treatment Program	As part of the pretreatment assessment at the Regional Treatment Centre Sexual Offender Treatment Program	M = 5.1 years	Canada
Marshall, Miller, Cortoni, and Helmus (2020)	New arrest or charge for a sexual offense	739	Female sex offenders released from prison	As part of the risk level assessment for the sex offender public registry	M = 67.0 years	USA
Martens, Rettenberger, and Eher (2015)	New sentence for any new sexual hands-on and hands-off offense	452	Male sex offenders released from prison	As part of a presentence screening for treatment planning	M = 5.8= years	Austria
Reeves, Ogloff, and Simmons (2018)	Reconviction for a sexual offense	502	Male sex offenders assessed by the Victorian Institute of Forensic Mental Health	As part of presentence assessments for the courts and Victorian Adult Parole Board	5 years	Australia
Smallbone and Rallings (2013)	Arrest for a sexual offense	399	Male sex offenders released from prison	As part of the routine screening protocols	M = 29 months	Australia
Tsao and Chu (2021)	New charge for a sexual offense	134	Male sex offenders on community supervision	As part of the risk assessment during presentencing to determine suitability for community supervision	M = 3.7 years	Singapore
Veith (2018)	New entry in North Dakota criminal database for a contact sexual offense	136	Male sex offenders from an outpatient treatment center	As part of the routine assessment or treatment evaluation	M = 51.3 months	USA

*Note.* M = average follow-up length

**Fig. 1:**
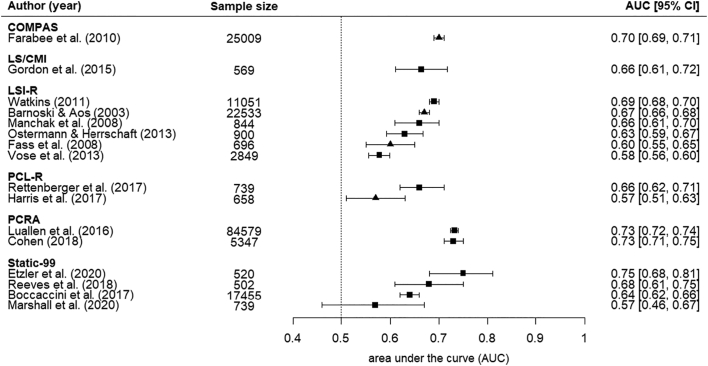
Area under the curve statistics for independent validation studies for risk assessment tools used at sentencing with sample sizes with more than 500. Note: ■ = 95% CI reported; ▲ = 95% CI estimated.

For our primary outcome, in independent validation studies with a sample size of more than 500 participants (k = 16, Fig. 1), the AUCs ranged from 0.57 to 0.75 (Fig. 1, Table 3). Most evidence was found for the LSI-R (k = 6), with AUCs ranging from 0.58 to 0.69. Four investigations examined the Static-99, two each the PCRA and the PCL-R, and there were single reports of COMPAS and LS/CMI. Results of pooled AUCs are presented in Table 3. The tool with the highest AUC was PCRA (i.e., 0.73), while all the other tools had a pooled AUC of equal to or less than 0.66. All but two (Cohen, 2018; Watkins, 2011) of these 16 studies had a high risk of bias based on PROBAST (**Supplementary** Table 1 and **Supplementary** Fig. 2). There was no clear evidence of publication bias when studies of all tools were included in the analysis (*z* = −0.73, *p* = 0.47).

**Table 3:** Selected discrimination estimates for independent validation studies with *N* > 500.

		Summary statistics		Pooled statistics	
Instruments	k	Mdn	IQR	AUC	95% CI
LSI-R	6	0.65	0.61–0.67	0.64	0.60–0.68
PCL-R	2	0.62	0.59–0.64	0.62	0.53–0.70
PCRA	2	0.73	0.73–0.73	0.73	0.73–0.74
Static-99	4	0.66	0.62–0.70	0.66	0.60–0.73

*Notes*: AUC = area under the curve (measure of predictive performance); k = number of studies; Mdn = Median AUC; IQR = interquartile range of AUCs; CI = confidence interval.

Random effects models were used to pool AUCs.

Results for COMPAS and LS/CMI not included because k = 1.

**Fig. 2:**
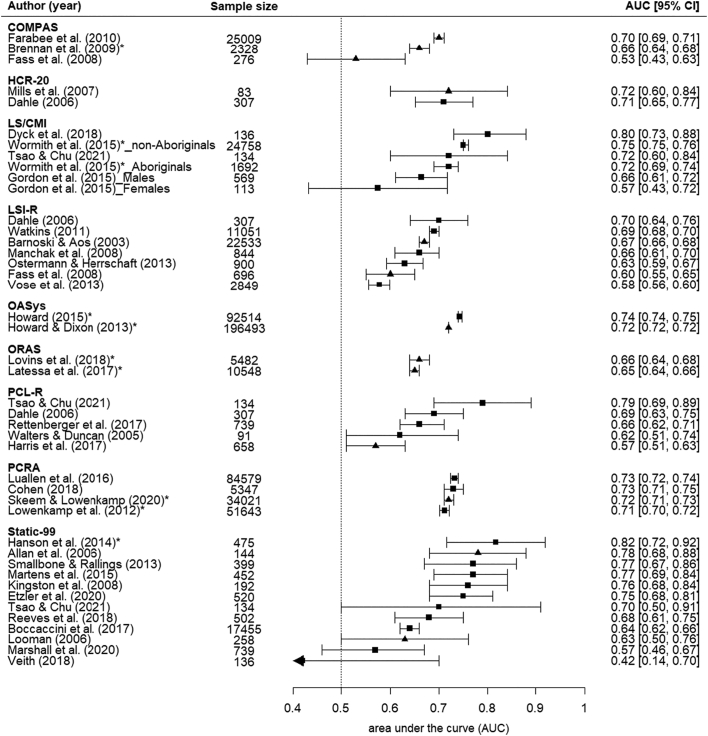
Area under the curve statistics for all validation studies for risk assessment tools used at sentencing (including non-independent and studies with small sample sizes). Ranked in order of AUC value. Note: * = non-independent validation study; ■ = 95% CI reported; ▲ = 95% CI estimated.

In a secondary analysis, we examined all independent studies irrespective of size. The number of studies investigating the Static-99 increased to 11 (from 4), and these typically reported higher AUCs than the larger studies (Fig. 2, **Supplementary** Fig. 3). Smaller studies also reported higher AUCs for LS/CMI.

We also summarised the performance of risk tools in non-independent validation studies (Fig. 2). For Static-99, tool developers were involved in a small study (with less 500 participants) which reported the highest AUC. In studies collecting data at the presentencing stage, most reported AUCs of 0.70 or higher (**Supplementary** Fig. 4).

In addition, we investigated the reliability of these tools (**Supplementary** Table 2). The most commonly reported outcome measure was the intra-class coefficient (ICC), a measure of interrater reliability, which ranged from 0.70 to 0.90. range The lowest interrater agreement was 0.68 for the total score of the LS/CMI. Concordance rates varied widely. We found only one relevant inter-reliability estimate for COMPAS, LS/CMI, and HCR-20, and none for OASys and PCRA.

## 4 Discussion

In this systematic review of 36 studies of risk assessment tools used to inform decision-making in the criminal justice system that followed up 597,665 participants for repeat offending, we identified performance measures for nine tools. In the 16 independent investigations with more than 500 participants each, the most common reported outcome statistic was the AUC, which ranged from 0.57 to 0.75.

Overall, the extent and quality of evidence in support of these tools is typically poor to moderate, based mainly on AUC values, and the reporting of other key performance measures such as true and false positive and negatives is inadequate. AUCs are only one measure of discrimination (which calculates the extent to which a tool separates out individuals with the outcome of interest from those without that outcome). However, reporting guidelines for prediction models recommend that other measures of discrimination are presented (such as true and false positives and negatives; Collins et al., 2015). For sentencing, this is particularly important as a key measure is the rate of false positives – as the implications of sentencing decisions can be harmful for the individual, such as a custodial (compared with a community) sentence, or a longer period of detention or community order. The ethical implications of false negatives are important as releasing potentially dangerous persons will have to be weighed up in terms of public safety and health (T. Douglas et al., 2017). The use of high quality risk assessment tools could also improve efforts towards decarceration and diversion of released prisoners to more productive activities and prioritize supervision and treatment of high-risk persons.

Another key performance measure for tools is calibration, which tests how well expected probabilities compare with observed ones. So if a tool estimates the risk of reoffending to be 10% in an individual, calibration tests how close this is to the actual reoffending rate. Only two of the 36 studies reported calibration, and these were all for one sexual reoffending tool. Calibration also applies to tools that provide risk categories rather than probability scores. For example, if a tool has two categories of <50% and ≥ 50%, then calibration tests whether these are accurate. Discrimination simply asks if the tool can separate out risk groups, but these groups could be based on very different actual risks. In other words, to take an example of a tool that uses a cut off of 50% for low/high risk, it may discriminate moderately well using this cut off – with most people who reoffend being in the higher risk bin, and most people who do not reoffend being the lower risk category. But the tool may be systematically be off target and the 50% cut off does not reflect actual reoffending risk. High risk may relate to an actual reoffending rate of 30% or 70% - we do not know using an AUC on its own. In theory, then, it is possible that a tool will have a perfect AUC of 1 but the high risk cut-off is inaccurate. Thus, AUCs have very limited utility in terms of their practical implications, and should be presented with other performance measures. Other work has shown poor calibration of one of the included tools, the PCRA, for younger and older age groups, and women in a probation sample (Monahan, Skeem, & Lowenkamp, 2017; Skeem, Monahan, & Lowenkamp, 2016).

Even based on AUCs, the most commonly used tool, the LSI-R, reported AUCs ranging from 0.58 to 0.69 in independent validation studies, lower than several recently developed tools. This means that the tools do clearly discriminate better than chance, and other work has shown that they typically are more accurate than unstructured human decision-making (Ægisdóttir et al., 2006). AUC estimates are higher in better quality tools in criminal justice, such as OxRec, where the external validation was found to have an AUC of 0.76 (Fazel et al., 2016) and the OxMIV tool when used for inpatient violence in a prison setting, with an AUC of 0.72 (Negatsch, Voulgaris, Seidel, Roehle, & Opitz-Welke, 2019). Comparisons with medicine, for example, where diagnostic and prognostic tools have been extensively researched is difficult as predictors and outcomes are different (Topol, 2019).

We found that there are no clear differences in AUCs between individual sentencing tools. There were only two studies of the PCRA that reported slightly higher AUCs than LSI-R and COMPAS. However, this is too limited an evidence base, given the small number of validations, to determine differences in tool performance, particularly in the absence of other measures of discrimination and calibration (DeLisi, Elbert, & Drury, 2018). In addition, research has reported that the AUCs of PCRA varied by reoffending outcomes, with a lower AUC in predicting sexual reoffending (0.63) than other types of recidivism (≥ 0.70; Cohen & Spidell, 2016). There were no differences in AUCs between structured professional judgement (SPJ) and actuarial tools. SPJ tools such as HCR-20 have been promoted as a benchmark. However, this review and previous studies have suggested that such SPJ tools should be limited to identifying low risk individuals and their use as key determinants of sentencing is not supported by systematic review evidence (Fazel et al., 2012; Tully, 2017).

Another finding of the current review is that smaller studies and non-independent studies typically reported higher AUCs. In relation to smaller studies, defined as testing less than 500 participants, this was most clearly seen with the Static-99 where 5 of the 12 highest AUCs were from these smaller investigations. The higher AUCs in non-independent studies underscore the importance of external validations that are sufficiently powered, and those that are also replicated independently to reduce risk of bias. Guidelines based on prognostic modelling recommend at least 100 outcome events in any replication (Steyerberg, 2018), which would suggest that most replications will need more than 500 persons (assuming at least 20% reoffending rates over 1 year). However, such independent replications have been rare as funding has been mostly from official governmental agencies, many of whom commission the tool developers to evaluate their performance. External funding organizations and research charities could play an important role here in funding high quality studies of the performance of these tools in practice. They are more likely to ensure that the scientific quality of proposals meets basic criteria.

This review has two major strengths. First, it followed a clear and focused search strategy to identify validation studies that were independent from the tool developers, and used for sentencing alone. This allows for a more real-world estimate of tool performance, particularly as we found further evidence of authorship bias. It also means that the reported estimates were not based on studies reporting selected samples that tend to overestimate performance, such as from high security settings and short-term institutional outcomes. Second, we collected data on all available quantitative measures of the predictive performance to allow for a more complete evaluation of performance. This has highlighted the lack of key performance measures in how these studies are reported.

We found a common set of limitations in the primary research. First, among the 11 commonly used sentencing risk assessment tools, only seven of them were externally validated without involvement of the tool developer. In addition, only four out of seven tools (LSI-R, PCL-R, Static-99, and LS/CMI) had more than two external validations. More such studies are needed to draw conclusion on the performance and generalizability of a particular tool (Fazel & Wolf, 2018). Second, very few included studies reported calibration performance and only one (Walters & Duncan, 2005) used a classification plot showing sensitivity and specificity conditional on risk thresholds (Verbakel et al., 2020). Such figures would allow comparing different models conditional on risk thresholds and also comparisons of performance across a range of cut-off scores within the same model. This would help criminal justice professionals and clinicians to use a tool based on what they think is the relevant threshold for a certain outcome and thereby increase the utility of the tool. The expected/observed (E/O) ratio, a key measure of calibration, was only reported in two out of 36 studies and only for Static-99, one of which was 1.9, indicating poor calibration. Third, the vast majority of studies had a high risk of bias in PROBAST (Wolff et al., 2019). This was mainly due to the quality of outcome data and analytical approach used in the validation studies. When considering whether the outcome was determined appropriately, high risk of bias was assigned if the outcome data were only collected for those who stayed in the same state/region but not for those who have moved during the follow up. This was a methodological limitation for most US-based validation studies (10 of the 16 independent investigations) as they usually only had access to the databases within a state. Outcome was rated as high risk of bias if predictors were not excluded from the outcome definition. As previous violent conviction is a strong predictor for reconviction, it was typically included in the prediction models for violence. In addition, eight out of 16 studies were rated as having a high risk of bias due to their analytical approach. The main analytic problems were not using appropriate methods to deal with varying follow-up periods (Rettenberger et al., 2017; Vose et al., 2013), not including all participants in the final analysis (Reeves et al., 2018), including additional predictors to the model (Gordon et al., 2015), not reporting confidence intervals of the reported AUC (Farabee et al., 2010; Fass et al., 2008), and having less than 100 outcome events (Etzler et al., 2020; Marshall et al., 2020). At the same time, PROBAST was developed for prediction models in medicine, and the threshold for bias may be too low for studies reporting crime outcomes (e.g. in the use of predictors for the outcome definition). There was substantial heterogeneity in outcome definitions and follow-up periods, which explains our decision in the primary analyses not to pool AUCs. As a secondary analysis, we used random-effects to pool AUCs, and the estimates were typically similar to the median AUCs. In addition, no subgroup or meta-regression was possible to examine factors associated with the predictive validity of the tools.

## 5 Conclusion

In this systematic review of external validation studies of 11 common risk assessment tools, most investigations solely reported the AUC as an indication of model performance, but did not present other key measures including rates of false positives and negatives, and calibration. As such, based on the current published evidence, the highest priority is for researchers to work towards addressing the key methodological limitations identified in previous work. Jurisdictions that are considering introducing such instruments for the first time should test them in independent validation studies as part of their implementation strategy. Their predictive performance will be one factor alongside scalability, transparency, and ethical issues.

## Funding

SF is funded by a Wellcome Trust Senior Research Fellowship (Grant no. 202836/Z/16/Z).

## Appendix A Supplementary data

Supplementary material Image 1
