# Supplementary material for: The predictive performance of criminal risk assessment tools used at sentencing: Systematic review of validation studies
Source: J Crim Justice. 2022 Jul-Aug;81:101902. doi: 10.1016/j.jcrimjus.2022.101902 (PMC9755051; doi:10.1016/j.jcrimjus.2022.101902)
Supplement: Supplementary file 1 — Supplementary material [file mmc1.docx]

**ssAppendix**

| 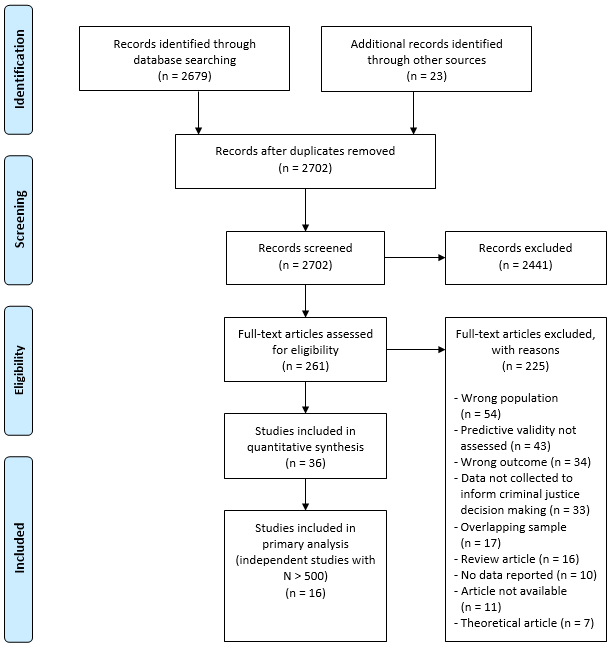 |
| --- |
| **Supplementary Figure 1.** PRISMA flowchart. |

| **Supplementary Table 1** - Risk of bias ratings for risk assessment studies used for sentencing | | | | | |
| --- | --- | --- | --- | --- | --- |
| **Study** | **Participants** | **Predictors** | **Outcome** | **Analysis** | **ROB: Overall** |
| Barnoski (2003) | ? | + | - | ? | High |
| Boccaccini (2017) | + | + | - | + | High |
| Cohen (2018) | + | + | + | + | Low |
| Etzler (2020) | + | + | + | - | High |
| Farabee (2010) | + | + | - | - | High |
| Fass (2008) | - | + | - | - | High |
| Gordon (2015) | - | + | + | - | High |
| Harris et al. (2017) | + | + | - | + | High |
| Luallen (2016) | + | + | - | + | High |
| Manchak (2008) | ? | - | - | ? | High |
| Marshall et al. (2020) | + | - | - | - | High |
| Ostermann (2013) | + | + | - | + | High |
| Reeves (2018) | + | + | - | - | High |
| Rettenberger et al. (2017) | + | + | + | - | High |
| Vose (2013) | - | + | - | - | High |
| Watkins (2011) | + | + | + | + | Low |
| *Note:* ROB = risk of bias; + = low ROB; - = high ROB; ? = ROB unclear. | | | | | |

| 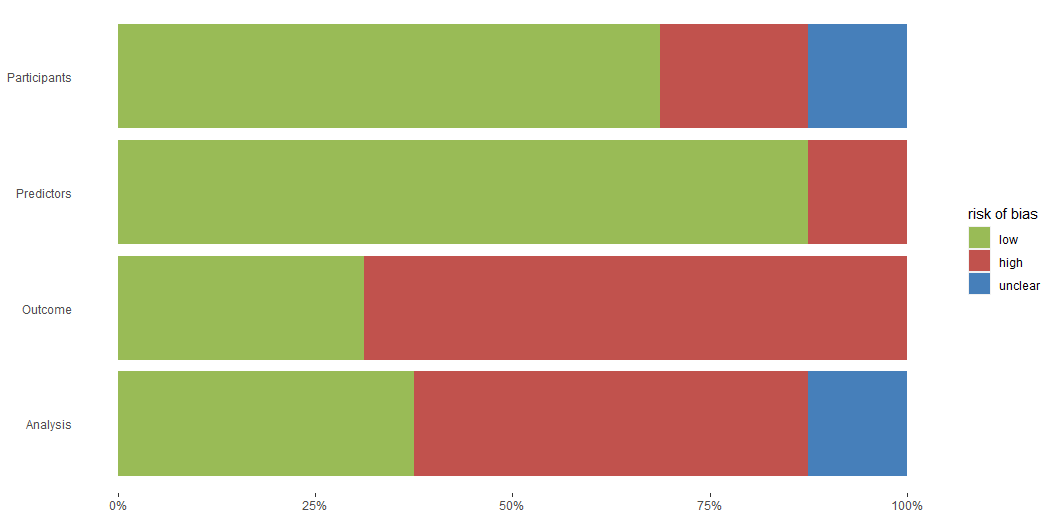 |
| --- |
| **Supplementary Figure 2**. Graphical representation of PROBAST ratings |

| **Supplementary Table 2** - **Discrimination and reliability estimates for all included studies** | | | | | | | |
| --- | --- | --- | --- | --- | --- | --- | --- |
|  | Outcome | | | | | | |
|  | AUC | | |  | Interrater reliability | | |
| Instruments | k | Mdn | IQR |  | k | Mdn | IQR |
| COMPAS | 3 | .66 | .60 - .68 |  | 1^†^ | - | - |
| HCR-20 | 2 | .72 | .71 - .72 |  | 1^†^ | - | - |
| LS/CMI | 6 | .72 | .68 - .74 |  | 1^†^ | - | - |
| LSI-R | 7 | .66 | .62 - .68 |  | 2 | .79 | .71 - .86 |
| OASys | 2 | .73 | .73 - .74 |  | - | - | - |
| ORAS | 2 | .66 | .65 - .66 |  | 1^†^ | - | - |
| PCL-R | 5 | .66 | .62 - .69 |  | - | - | - |
| PCRA | 4 | .73 | .72 - .73 |  | - | - | - |
| Static-99 | 12 | .73 | .64 - .77 |  | 3 | .79 | .79 - .85 |
| *Notes*: Interrater reliability measures include ICC, Pearson’s *r.*  *AUC* = area under the curve (measure of predictive performance); k = number of studies; Mdn = Median; IQR = Interquartile range.  † = median and IQR not reported since k = 1. | | | | | | | |

| 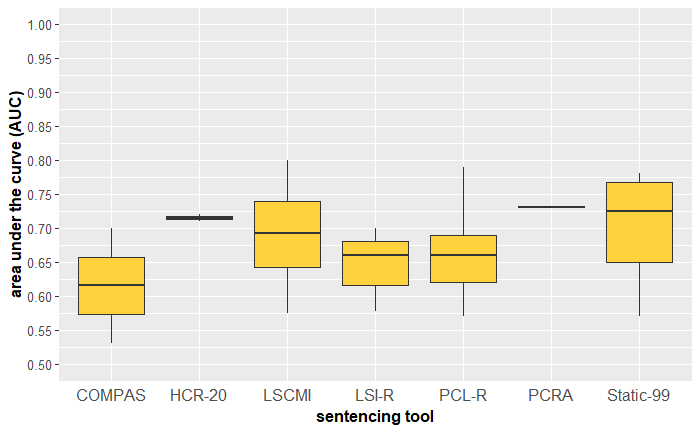 |
| --- |
| **Supplementary Figure 3.** Area under the curve statistic for all independent risk assessment tools (irrespective of size).  Box plots indicate the interquartile range of AUCs with the horizontal line showing the median. |


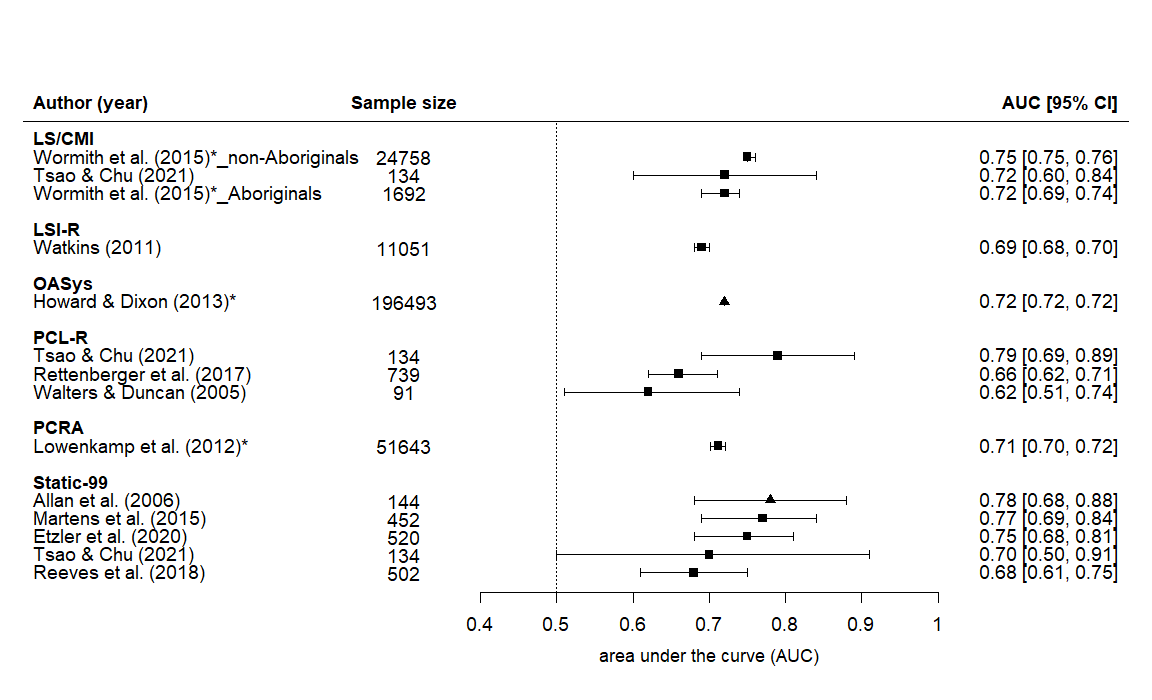


| **Supplementary Figure 4.** Area under the curve statistic for studies using tools specifically at the presentencing stage |
| --- |
